# Acceptance and commitment therapy in a psychiatric day hospital—A longitudinal naturalistic effectiveness trial

**DOI:** 10.3389/fpsyt.2022.1052874

**Published:** 2023-01-11

**Authors:** Ronja Rutschmann, Nina Romanczuk-Seiferth, Christoph Richter

**Affiliations:** ^1^Clinic for Psychiatry, Psychotherapy and Psychosomatics, Vivantes Klinikum Kaulsdorf, Berlin, Germany; ^2^Charité – Universitätsmedizin Berlin, Corporate Member of Freie Universität Berlin and Humboldt-Universität zu Berlin, Department of Psychiatry and Neurosciences, Berlin, Germany

**Keywords:** Acceptance and Commitment Therapy, transdiagnostic, naturalistic setting, reliable change, quality of life, general functioning, psychiatric day hospital

## Abstract

**Objectives:**

Despite the transdiagnostic approach and the good cross-professional applicability, only few studies have examined the effects of Acceptance and Commitment Therapy (ACT) in a naturalistic clinic setting. This study aims to help closing this gap by investigating the effects of ACT in a psychiatric day hospital during COVID pandemic. It was investigated whether psychopathological symptomology decreased, and quality of life and general functioning improved with the treatment. Additionally, longitudinal effects were tested.

**Methods:**

Participants in this follow-up-design were 92 patients (64.1% female) of a psychiatric day hospital. Survey data of clinical symptoms, quality of life and global functioning were assessed at three time points (with admission, discharge, and 3 months after treatment). Differences between time points were tested using two-sided paired samples *t*-tests. Additionally, the reliability of change index (RCI) was calculated.

**Results:**

From pre-treatment to post-treatment, symptomology decreased significantly (*d* = 0.82–0.99, *p* < 0.001), and global functioning as well as quality of life increased significantly (*d* = 0.42–1.19, *p* < 0.001). The effects remained relatively stable, with no significant change between post-treatment and follow-up. The difference between pre-treatment and follow-up was significant for clinical symptoms, physical and psychological wellbeing, and global quality of life (*d* = 0.43–0.76, *p* < 0.007).

**Conclusion:**

The significant and sustained improvement in all measures indicates that patients are benefiting from the treatment. Since the trial was neither randomized nor controlled, effects have to be interpreted with caution. Possible influences of the pandemic are discussed.

**Clinical trial registration:**

http://www.drks.de/DRKS00029992, identifier DRKS00029992.

## 1. Introduction

Psychiatric day hospitals have become more widespread in recent years (1). They have proven to be as effective as inpatient treatment in improving symptomology and quality of life and even more effective with respect to social functioning (2). At the same time, overall costs of the treatment are significantly lower for psychiatric day hospitals (3, 4) even though patients receive a larger number of more expensive therapies there (1). A therapeutic approach that is very suitable for use in psychiatric day hospitals, is Acceptance and Commitment Therapy (ACT) (5).

ACT combines classical behavioral therapy techniques with mindfulness and acceptance-based strategies as well as methods of value-based action. ACT is a transdiagnostic procedure and has become increasingly popular in the last years due to its proven effectiveness and applicability in various settings (6). A basic assumption of ACT is that mental disorders are significantly related to psychological inflexibility, i.e., “the inability to persist or change in the service of long term valued ends” (7). The link between psychological inflexibility and psychopathology has been demonstrated for a wide range of mental disorders. Therefore, psychological inflexibility is considered an important transdiagnostic factor of mental illness (8). The aim of ACT is to develop a more flexible way of dealing with thoughts and feelings in order to live a self-determined, meaningful, and vital life. This is achieved by working on six core processes: acceptance, being present, cognitive defusion, self as context, values, and committed action (7). Symptom reduction is not the primary goal, but rather a by-product of increased psychological flexibility (9, 10).

There are currently over 900 randomized controlled trials (RCT) that have demonstrated the efficacy of ACT (6), mostly defined and measured as symptom reduction. Some studies have shown that also the subjective quality of life improves significantly as a result of treatment with ACT [e.g., (11–13)]. One important limitation of RCTs is that they rarely reflect the everyday clinical routine. Inclusion criteria for participants are often very strict (e.g., one specific diagnosis, no comorbidities, and no drug abuse), while in naturalistic settings patients are often much more diverse. Additionally, in RCTs treatment is often strongly standardized and performed only by psychologists and medical doctors, instead of an individualized and multi-professional application as in clinical routine. It is important to note that this lack of studies in naturalistic settings applies not only to ACT, but to psychotherapy in general, and is even more striking in day hospitals (14).

Despite the transdiagnostic approach and the good cross-professional applicability of ACT, for a long time no study has tested the effectiveness of ACT in a transdiagnostic and interdisciplinary clinical setting such as a psychiatric day hospital. Little studies on this subject have been published just recently (14, 15). To date, however, none of these studies has reported longitudinal effects of ACT treatment in such a setting. In summary, there is a lack of evaluations of ACT in real-world settings, such as a transdiagnostic clinical setting, especially regarding follow-up data. The present study is intended to help closing this gap. Effects of ACT-based treatment were tested in an psychiatric day hospital with patients having different diagnoses, often comorbidities, and coming from very different sociodemographic backgrounds. Additionally, the study took place during a pandemic situation, adding further challenges to the typical real-life mental health inpatient treatment. The aim of the study was to test the impact of ACT on psychopathology, quality of life, and general functioning in a psychiatric day hospital.

## 2. Materials and methods

### 2.1. Procedure

The Ethics Committee of the Medical Association Berlin approved the research project (12 February 2020, case number Eth-03/20). All participants included in the study gave their written informed consent. Participants were recruited in a general psychiatric day hospital in Germany, where ACT had been implemented as therapeutic concept about a year before. Starting February 2020, all patients of the psychiatric day hospital received a set of questionnaires on their admission day (pre-treatment) and on their discharge day (post-treatment). Additionally, the respective therapist who conducted the admission and discharge interviews, estimated the individual global functioning at the respective time. Participants who agreed to the follow-up, received another set of questionnaires 3 months after their release *via* mail.

### 2.2. Participants

**FIGURE 1:**
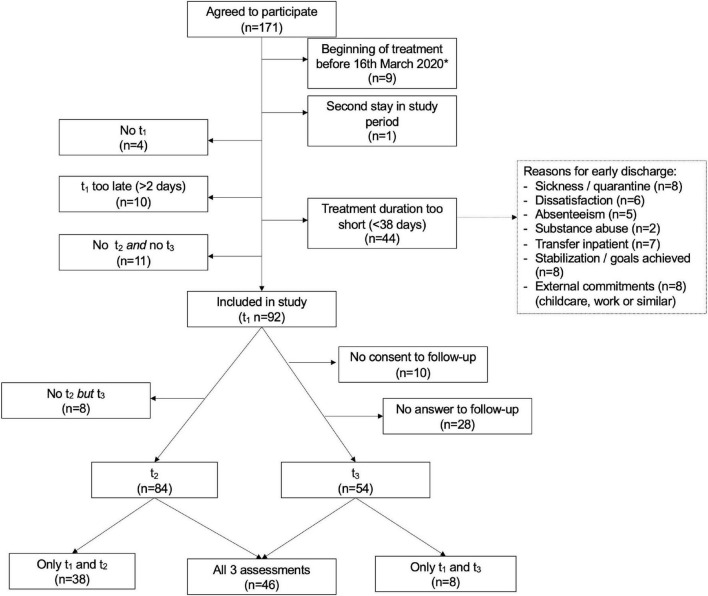
Flow of participants throughout the study. t_1_, pre-treatment assessment; t_2_, post-treatment assessment; t_3_, follow-up assessment. *16 March 2020: Beginning of Covid-related restrictions in Germany.

The initial sample consisted of 171 patients who agreed to participate. Inclusion criteria were (1) filling out the questionnaires in time (i.e., within 2 days from admission), (2) minimum treatment duration of 38 days (that means five and a half weeks to be able to work on all six core processes of ACT). The data collection started just before the COVID outbreak in Europe. In order to keep the impact of COVID restrictions as equal as possible for all participants in this study, only (3) patients who were admitted to the psychiatric day hospital after the start of the COVID restrictions in Germany (16 March 2020) were included in the study. For a detailed flow of participants (see Figure 1).

A total of 92 participants met inclusion criteria and were included in the study. Their mean age was 41.28 years (*SD* = 13.34, range 18–65) and the mean duration of treatment in the psychiatric day hospital was 48.35 days (*SD* = 6.71, range 38–65). Sample characteristics are depicted in Table 1.

**TABLE 1:** Characteristics of participants.

		N	%
**Gender**			
	Male	33	35.9%
	Female	59	64.1%
**Highest level of education**			
	None	2	2.2%
	Secondary School (“Hauptschulabschluss”)	14	15.2%
	Intermediate (“Realschulabschluss”)	53	57.6%
	A-levels (“Abitur”)	14	15.2%
	University degree	8	8.7%
	unknown	1	1.1%
**Main diagnosis**			
F2	Schizophrenia, schizotypal, and delusional disorders	8	8.7%
F3	Mood (affective) disorders	55	59.8%
F4	Neurotic, stress-related, and somatoform disorders	24	26.1%
F6	Disorders of adult personality and behavior	5	5.4%
**Secondary diagnosis**			
F1	Mental and behavioral disorders due to psychoactive substance use	9	9.8%
F2	Schizophrenia, schizotypal, and delusional disorders	1	1.1%
F3	Mood (affective) disorders	9	9.8%
F4	Neurotic, stress-related, and somatoform disorders	13	14.1%
F5	Behavioral syndromes associated with physiological disturbances and physical factors	3	3.3%
F6	Disorders of adult personality and behavior	7	7.6%
	None	50	54.3%

Diagnoses were classified according to the International Statistical Classification of Diseases and Related Health Problems 10th Revision (ICD-10) (29).

### 2.3. Acceptance and Commitment Therapy

The therapeutic program in the psychiatric day hospital was based on the six key processes of ACT (see above). These processes were addressed across diagnoses and therapeutic professions with a different core process being the focus of treatment each week. The therapies included group psychotherapy (twice a week). occupational and art therapy (two to three times a week), music therapy (once a week by prescription), movement therapy (twice a week), mindfulness training (three times a week), individual psychotherapy (once a week), and an ACT Matrix group (every other week). Every group therapy session lasted 50 min, individual psychotherapy 25 min.

Group psychotherapy was based on the manual “Therapie-Tools Akzeptanz- und Commitmenttherapie” (16). The core process of each week was addressed with exercises, worksheets, and homework. The Matrix group was led by a psychiatric nurse who assisted patients to complete their individual matrix model and reflect it [see Polk et al. (17)].

The professional team consisted of psychologists, psychiatrists, psychiatric nurses, social workers, occupational therapists, movement therapists, and music therapists. All had attended several days of in-house training on ACT. The psychologists, psychiatrists, and some of the psychiatric nurses had also participated in external training on ACT. All participated regularly in an external ACT-based supervision.

Patients attended the psychiatric day clinic 5 days a week (Monday to Friday). Normally, treatment time is 7:30–15:00. Treatment conditions were adjusted to the COVID pandemic situation. Thus, in order to keep the number of contacts low while continuing to provide care for enough patients, the groups were divided and attended the psychiatric day hospital either in the morning (7:30–11:00) or in the afternoon (11:30–15:00). However, it was always ensured that ACT group psychotherapy (twice a week) and individual therapy took place for all participants.

### 2.4. Materials

Materials were the same at pre-treatment and post-treatment. Diagnoses, potential limitations for the study, and the individual global functioning were documented by the therapist conducting admission and discharge respectively. Furthermore, the participants received a battery of standardized questionnaires. At follow-up, patients also received a set of questionnaires *via* mail (self-report measures).

Global functioning was assessed *via* the *Global Assessment of Functioning Scale* (GAF) (18), a diagnostic screening in the form of an expert rating. The scale measures the psychosocial functioning level of a person regardless of the severity of the symptoms.

The Global Severity Index (GSI) of the *Symptom Checklist-90-Standard* (SCL-90-S) (19) was used to assess the current psychopathological symptomology.

The *Beck Depression Inventory-II* (BDI-II) (20) was used to assess the depressive mood of the participants. Despite the transdiagnostic approach of this study, a disorder-specific questionnaire was used here for three reasons. First, the BDI-II is often used for research, which allows a comparison of the results. The second reason is the high incidence and comorbidity of depression with other mental disorders, often depression being the subsequent diagnosis (21). Third, it has been demonstrated that the BDI-II is not only suitable for the measurement of clinical depression, but also for the assessment of subclinical depressive symptoms (22).

Subjective quality of life (QL) was assessed *via World Health Organization Quality of Life—Short Version* (WHOQOL-BREF) (23). The 26 items of the questionnaire record the subjectively perceived quality of life in the dimensions of physical wellbeing, psychological wellbeing, social relationships, environment, and global quality of life.

In addition, it was surveyed whether medication was changed during the treatment at the psychiatric day hospital, in order to detect possible confounding factors.

### 2.5. Data analysis

Differences between dropouts and the final sample were tested using two-sided independent *t*-tests for interval-scaled variables and chi-squared tests for categorical variables (IBM SPSS Statistics Version 28.0.1.0, RRID: SCR_019096). Missing data in the final sample was analyzed using Little’s MCAR test. GAF-scores of different raters were contrasted *via* ANOVA to reveal a possible rater bias.

Pre-post- and pre-follow-up-differences in study variables were tested for statistical significance using two-sided paired samples *t*-tests. The significance threshold was adjusted using the Bonferroni method, from *p* < 0.05 to *p* < 0.006 for pre-post comparisons and to *p* < 0.007 for pre-follow-up comparisons. Cohen’s *d* was used to calculate effect sizes and Cohen’s standard was used to interpret them (*d* ≥ 0.20 small, *d* ≥ 0.50 medium, *d* ≥ 0.80 large effect).

Reliability of change was calculated based on the reliable change index (RCI) according to Jacobson and Truax (24). The RCI is a threshold for reliable change, i.e., a change that is unlikely to occur due to an inaccurate measurement alone. Cases with a positive change greater than the calculated RCI were considered reliable improvements. Simultaneously, cases with a negative change greater than the RCI were considered reliable deterioration. The RCI for each measure was calculated with Excel (Version 16.61.1), based on the Zahra (25) RCI calculator. Reliability measures were retrieved from the literature. Because no reliability is reported for the dimension of global quality of life in the WHOQOL-BREF, the RCI could not be calculated for that scale.

The possible influence of change of medication was tested *via* a 3 × 4 [time (pre, post, follow-up) × medication (unchanged, decreased/stopped, switched, increased/applied)] repeated-measures ANOVA. Since there was no GAF-value at the time of follow-up, a 2 × 4 ANOVA was used here.

## 3. Results

### 3.1. Basic data analysis

The dropouts did not differ significantly from the final sample in age, main diagnosis, or in the variables BDI, GSI, or any of the scales of the WHOQOL-BREF (all *p* > 0.05). They did differ, though, in the GAF-rating, with dropouts scoring significantly lower than the final sample (*M*_*diff*_ = 3.88, *p* = 0.004). The ANOVA showed no significant difference in the average GAF-rating between raters (*p* = 0.995). Missing data in the final sample showed to be missing completely at random (Little-Test, all *p* > 0.05), therefore pair-wise deletions were applied. This leads to different sample sizes across analyses. No significant interaction could be found between time and change of medication for any of the study variables (all *p* > 0.33).

### 3.2. Changes in symptomology and global functioning

Symptomology, as measured by GSI and BDI-II, decreased significantly from pre-treatment to post-treatment. The global functioning as estimated *via* GAF increased significantly. All effects were large (see Table 2). Similar effects could be found for the comparison between pre-treatment and follow-up. Symptomology decreased significantly; all effects were of medium size (see Table 3). There was no significant change in symptomology between post-treatment and follow-up (see Table 4).

**TABLE 2:** Contrast of pre-treatment with post-treatment in study variables.

	Pre-treatment		Post-treatment		Paired *t*-test						
Variable	*M*	*SD*	*M*	*SD*	*M*Δ	*SD*	*95% CI*	*t*	*df*	*d*	
BDI	28.26	12.80	16.99	11.78	11.28	11.34	[8.75; 13.80]	8.89	79	0.99	**
GSI	66.16	6.88	60.66	8.98	5.50	6.69	[4.01; 6.99]	7.36	79	0.82	**
GAF	50.66	7.95	62.40	10.24	-11.74	9.87	[−9.54; -10.64]	-10.64	80	-1.19	**
**QL**											
Psy.	40.04	17.44	53.37	18.42	-13.32	16.89	[−17.01; −9.64]	-7.19	82	-0.79	**
Soc.	51.44	19.73	58.80	20.09	-7.36	17.71	[−11.28; −3.44]	-3.74	80	-0.42	**
Phys.	47.30	13.72	60.04	15.89	-12.74	13.87	[−15.80; −9.67]	-8.27	80	-0.92	**
Env.	61.77	14.91	76.31	14.95	-5.54	9.80	[−7.68; −3.40]	-5.15	82	-0.57	**
Glob.	38.12	18.32	51.70	17.09	-13.58	19.59	[−17.91; −9.25]	-6.24	80	-0.69	**

CI, confidence interval. Higher values in BDI and GSI represent higher symptom severity. Higher values in GAF represent higher general functioning and higher values in QL represent higher subjective quality of life. **p < 0.001.

**TABLE 3:** Contrast of pre-treatment with 3 months follow-up in study variables.

	Pre-treatment		Follow-up		Paired *t*-test						
Variable	*M*	*SD*	*M*	*SD*	*M*Δ	*SD*	*95% CI*	*t*	*df*	*d*	
BDI	30.46	13.72	21.04	17.05	9.44	12.48	[5.95; 12.90]	5.44	51	0.76	**
GSI	66.92	7.36	60.71	12.26	6.21	9.72	[3.51; 8.92]	4.61	51	0.64	**
**QL**											
Psy.	39.83	18.93	50.39	24.84	-10.57	19.83	[−16.03; −5.10]	-3.88	52	-0.53	**
Soc.	53.69	18.33	57.37	21.81	-3.69	21.73	[−9.74; 2.36]	-1.22	51	-0.17	
Phys.	46.62	13.85	54.81	20.73	-8.20	19.29	[−13.62; −2.77]	-3.04	50	-0.43	*
Env.	64.69	12.92	67.63	17.62	-2.94	11.57	[−6.13; 0.25]	-1.85	52	-0.25	
Glob.	37.74	16.88	49.76	23.15	-12.02	22.00	[−18.14; −5.90]	-3.94	51	-0.55	**

CI, confidence interval. Higher values in BDI and GSI represent higher symptom severity. Higher values in QL represent higher subjective quality of life. *p < 0.007 (Bonferroni-corrected), **p < 0.001.

**TABLE 4:** Contrast of post-treatment with 3 months follow-up in study variables.

	Post-treatment		Follow-up		Paired *t*-test					
**Variable**	***M***	***SD***	***M***	***SD***	***M* **Δ****	***SD***	***95% CI***	***t***	***df***	***d***
BDI	17.23	13.63	19.95	17.70	-2.72	13.32	[−6.82; 1.38]	-1.34	42	-0.20
GSI	60.82	10.32	59.64	12.64	1.18	8.46	[−1.39; 3.75]	0.93	43	0.14
**QL**										
Psy.	54.38	19.48	52.37	25.53	2.01	18.43	[−3.60; 7.61]	0.72	43	0.11
Soc.	58.62	16.96	57.20	22.13	1.42	18.33	[−4.15; 6.99]	0.51	43	0.22
Phys.	59.50	18.21	55.43	21.98	4.07	18.10	[−1.44; 9.57]	1.49	43	0.23
Env.	70.95	14.18	68.68	17.18	1.42	18.33	[−4.15; 6.99]	1.42	43	0.22
Glob.	51.45	18.74	50.00	23.62	1.45	19.52	[−4.55; 7.46]	0.49	42	0.07

CI, confidence interval. Higher values in BDI and GSI represent higher symptom severity. Higher values in QL represent higher subjective quality of life (all *p* > 0.14).

### 3.3. Changes in perceived quality of life

The subjective quality of life (QL) as measured by the WHOQOL-BREF increased significantly from pre-treatment to post-treatment in all dimensions, the smallest effect being reported in social relationships and the largest effect in physical wellbeing (see Table 2).

From pre-treatment to follow-up, QL increased significantly in the dimensions of physical wellbeing, psychological wellbeing, and global quality of life. No significant change could be found in the dimensions of social relationships and environment (see Table 3). QL did not change significantly between post-treatment and follow-up (see Table 4).

### 3.4. Reliability of change

**FIGURE 2:**
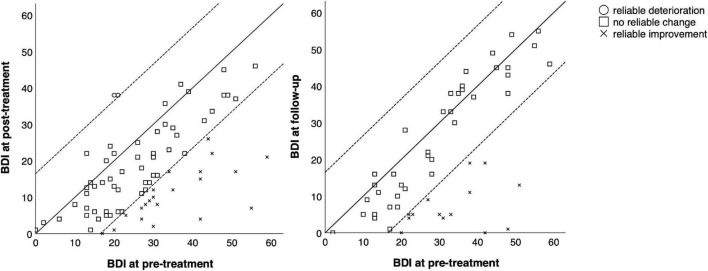
Comparison of pre-treatment, post-treatment, and follow-up scores in the BDI for each participant. Reliable change index was calculated according to Jacobson and Truax (24).

**FIGURE 3:**
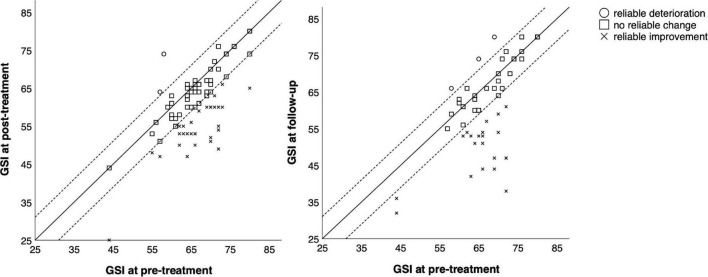
Comparison of pre-treatment, post-treatment, and follow-up scores in the GSI-scale for each participant. Reliable change index was calculated according to Jacobson and Truax (24).

**FIGURE 4:**
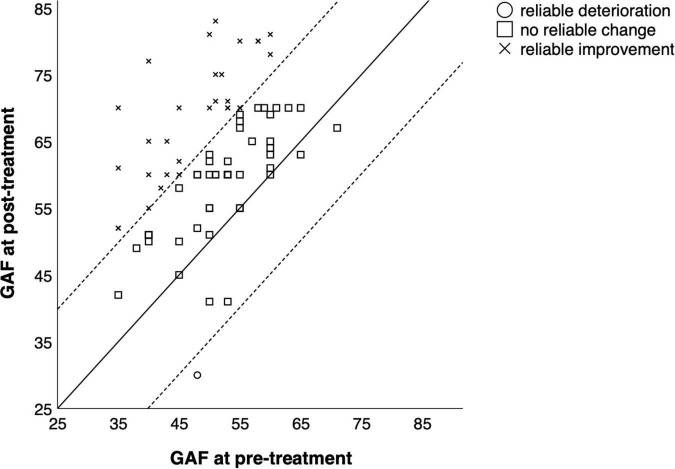
Comparison of pre-treatment and post-treatment scores in the GAF for each participant. Reliable change index was calculated according to Jacobson and Truax (24).

Detailed information on reliable changes in the individual measures can be found in Table 5. Figures 2–4 show the individual change of each participant as well as their categorization (reliable improvement, no reliable change, and reliable deterioration) in the measures BDI-II, GSI and GAF.

**TABLE 5:** Reliable change from pre-treatment to post-treatment and follow-up.

			Post-treatment			Follow-up		
				Deterioration	Improvement		Deterioration	Improvement
Variable	r_tt_^a^	RCI	n	n (%)	n (%)	n	n (%)	n (%)
BDI	0.78	16.52	80	2 (2.5%)	23 (28.7%)	52	0 (0.0%)	14 (26.9%)
GSI	0.90	6.04	80	2 (2.5%)	30 (37.5%)	52	3 (5.8%)	21 (40.4%)
GAF	0.56	14.86	80	1 (1.3%)	30 (37.5%)	–	–	–
**QL**								
Psy.	0.72	25.51	83	2 (2.4%)	13 (15.7%)	53	0 (0.0%)	12 (22.6%)
Soc.	0.87	27.08	81	1 (1.2%)	13 (16.0%)	52	2 (3.8%)	6 (11.5%)
Phys.	0.66	22.74	81	0 (0.0%)	17 (21.0%)	51	1 (0.9%)	8 (15.7%)
Env.	0.76	14.64	83	3 (3.6%)	15 (18.1%)	53	3 (5.7%)	10 (18.9%)

^a^ Reliability retrieved from literature: BDI (30), GSI (19), GAF (31), quality of life (32).

In total, 55 out of 84 participants (65.5%) showed reliable improvement from before to after treatment in at least one measure, and seven participants (8.3%) showed reliable deterioration in at least one measure. From pre-treatment to follow-up, 24 out of 54 participants (44.4%) showed reliable improvement and eight (14.8%) showed reliable deterioration in at least one measure.

## 4. Discussion

### 4.1. Key findings and interpretation

This study evaluated the effects of Acceptance and Commitment Therapy (ACT) in a naturalistic clinical setting—in a transdiagnostic psychiatric day hospital during the global COVID pandemic. As predicted, the symptomology decreased significantly from pre-treatment to post-treatment. The effects were large. These results are in line with other recently published studies, indicating that, also in a real-world clinical setting, ACT can be a very effective therapy (14, 15). The present study complements the previous findings with longitudinal data, confirming that the positive effects continue to persist after the treatment ends. This is remarkable, considering that in the same time, due to the COVID pandemic and associated restrictions, the average mental wellbeing as well as subjective quality of life decreased significantly in Germany (26–28). The treatment in the psychiatric day hospital does not only seem to compensate for this tendency, but even counteract it. Nevertheless, the pandemic situation also had an impact on the participants in this study. Several participants left handwritten notes on the questionnaires, emphasizing that the answers were influenced by the current pandemic situation. For example, several patients stated that they were afraid of crowds because of the fear of getting infected (referring to an item of the SCL-90 that is supposed to survey agoraphobia). That means that some GSI values might be amplified by the pandemic situation. Additionally, due to necessary contact limitations, therapy times for each patient were lower compared to pre-pandemic standards. This is also one possible explanation why the reliable improvement rates, though still good, were lower than in another similar study that investigated the effect of ACT in a transdiagnostic psychiatric day hospital (14).

The deterioration rates are to be considered critically, especially the percentage of deterioration between pre-treatment and follow-up and particularly in the domains of subjective quality of social relations and environment. One possible explanation is that there are patients that did not profit at all from the treatment. However, a closer look shows that, while improvement was more global, mainly affecting multiple measures, deterioration happened mostly in only one measure. In addition, the quality of social relationships as well as environment (which includes, for example, leisure opportunities) are likely to be particularly affected by COVID-related restrictions. Patients had very limited opportunities to work on improving the quality of social relationships and recreation time—which is usually an important component of the ACT key processes “values” and “committed action.”

As stated earlier, the primary goal of ACT is not symptom reduction, but the ability to live a self-determined and meaningful life (9, 10). Therefore, this study also examined how subjective quality of life and overall level of functioning changed with treatment. Both quality of life and global functioning increased significantly from pre-treatment to post-treatment. The effect for global functioning was large, the effects for quality of life were medium to large, depending on the respective dimension. These effects, too, remained relatively stable after the treatment with no significant difference between post-treatment and follow-up scores. Nevertheless, for the scales social relations and environment the effect of pre-treatment to follow-up was no longer significant. It is striking that after the treatment the quality of life was improved above all in the areas that can be located more internally (physical, psychological, and global quality of life) and less the external areas (social relationships and environment). One explanation might be, that ACT affects above all the appraisal of how one perceives oneself and not so much how one shapes the world around him. Since committed action is one core process of ACT, it might be falling short of its own expectations here. Another possible explanation, though, is that those areas are also the most difficult to change and sometimes even out of one’s own control. This is even more the case in a pandemic situation which results in contact restrictions and limited leisure opportunities, as already mentioned above.

### 4.2. Limitations and future directions

The present study has several limitations, mostly deriving from the fact that the study was conducted in a real-world setting during the COVID pandemic. The influence of the pandemic situation on the results is not clear. The previously described reduced wellbeing and quality of life in the general population during the pandemic did presumably apply also to the participants in this study. This may have attenuated the effects of treatment. On the other hand, the psychiatric day hospital might have been the only possibility for social contact for some patients at that time, which may have contributed to an improvement in symptomatology and quality of life during their stay. In addition, everyday clinical life was also affected by the pandemic, including a significantly reduced quantity of therapy for each patient. Additionally, patients had to be regularly tested for COVID and wear masks at all times. This led to feelings of incomprehension or frustration among some patients. Other patients were very anxious about the current situation and feared infection. All this could be an important factor influencing the results. As shown in the flowchart (Figure 1) the pandemic was also responsible for a large proportion of dropouts, for example, due to quarantine, infection, or canceled child care.

Additionally, due to the study design, the positive effects cannot be clearly attributed to the therapy rather than external effects or natural improvement of symptoms. Due to ethical as well as practical reasons though, it was not possible to include a waiting group or a TAU group in this specific setting. Even assuming that the positive effects are due to the therapy, this may be an indication of the effectiveness of a day clinic in general and not necessarily a result of ACT.

Another limitation is that the dropout rates were relatively high. It is important to emphasize that, unlike for example RCT studies, the sample was barely preselected. Additionally, the reasons for dropouts were very diverse, including quarantine, external commitments and even stabilization of psychopathology. Therefore, the dropouts do not reflect dissatisfaction or non-response.

Despite these limitations, studies in such naturalistic settings offer a wide range of advantages, since they reflect the applicability of a treatment in the real world. Future studies could try to include TAU groups in such a naturalistic setting and incorporate more follow-ups. Additionally, longitudinal studies should investigate the impact of the COVID pandemic on therapy outcomes. Another interesting question would be whether deterioration of the subjective quality of social relationships and environment could be a possible unwanted side-effect of the treatment. Additionally, in order to survey the impact of the pandemic situation, it would be interesting to repeat the study under more normal circumstances.

## Data availability statement

The raw data supporting the conclusions of this article will be made available by the authors, without undue reservation.

## Ethics statement

The studies involving human participants were reviewed and approved by Ethics Committee of the Medical Association Berlin (Ethik-Kommission bei der Ärztekammer Berlin). The patients/participants provided their written informed consent to participate in this study.

## Author contributions

RR performed material preparation, data collection and analysis, and wrote the first draft of the manuscript. All authors contributed to the study conception and design, commented on previous versions of the manuscript, and read and approved the final manuscript.
